# Structural hippocampal network alterations during healthy aging: a multi-modal MRI study

**DOI:** 10.3389/fnagi.2013.00084

**Published:** 2013-12-05

**Authors:** Amandine Pelletier, Olivier Periot, Bixente Dilharreguy, Bassem Hiba, Martine Bordessoules, Karine Pérès, Hélène Amieva, Jean-François Dartigues, Michèle Allard, Gwénaëlle Catheline

**Affiliations:** ^1^University of Bordeaux, INCIA, UMR 5287Talence, France; ^2^CNRS, INCIA, UMR 5287Talence, France; ^3^EPHEBordeaux, France; ^4^CHU de Bordeaux, Service de Médecine NucléaireBordeaux, France; ^5^RMSB, UMR 5536Bordeaux, France; ^6^Université de Bordeaux, ISPED, Centre ISPED, INSERM U 897Bordeaux, France

**Keywords:** hippocampal atrophy, limbic system, fornix, cingulum, DTI, healthy aging, mode of anisotropy

## Abstract

While hippocampal atrophy has been described during healthy aging, few studies have examined its relationship with the integrity of White Matter (WM) connecting tracts of the limbic system. This investigation examined WM structural damage specifically related to hippocampal atrophy in healthy aging subjects (*n* = 129), using morphological MRI to assess hippocampal volume and Diffusion Tensor Imaging (DTI) to assess WM integrity. Subjects with Mild Cognitive Impairment (MCI) or dementia were excluded from the analysis. In our sample, increasing age was significantly associated with reduced hippocampal volume and reduced Fractional Anisotropy (FA) at the level of the fornix and the cingulum bundle. The findings also demonstrate that hippocampal atrophy was specifically associated with reduced FA of the fornix bundle, but it was not related to alteration of the cingulum bundle. Our results indicate that the relationship between hippocampal atrophy and fornix FA values is not due to an independent effect of age on both structures. A recursive regression procedure was applied to evaluate sequential relationships between the alterations of these two brain structures. When both hippocampal atrophy and fornix FA values were included in the same model to predict age, fornix FA values remained significant whereas hippocampal atrophy was no longer significantly associated with age. According to this latter finding, hippocampal atrophy in healthy aging could be mediated by a loss of fornix connections. Structural alterations of this part of the limbic system, which have been associated with neurodegeneration in Alzheimer's disease, result at least in part from the aging process.

## Introduction

The limbic system is considered as the cerebral network devoted to memory function and it has been extensively studied in various neurological diseases, and particularly in age-related dementia. Assuming that the hippocampus is the key component of the limbic system, it can be hypothesized that a direct link exists between hippocampal neural injury and damage to White Matter (WM) bundles of this system. Two main connecting tracts in the limbic system are the cingulum bundle which constitutes the principal afferent pathway of the hippocampus, and the fornix which constitutes the principal efferent pathway of the hippocampus. The direct relationship between hippocampal injury and WM damage in these two bundles has been previously reported in Alzheimer's disease (AD) but the results of these investigations are somewhat inconsistent. Some studies have shown no evidence for a relationship between hippocampal atrophy and disruption of the cingulum bundle (Firbank et al., 2007; Nakata et al., 2009), whereas others have highlighted an association between atrophy of the hippocampus and integrity of the cingulum bundle (Xie et al., 2005; Villain et al., 2008; Choo et al., 2010; Villain et al., 2010; Agosta et al., 2011). A recent investigation demonstrated that a part of structural changes observed in the parahippocampal cingulum were independent of hippocampal atrophy (Salat et al., 2010). Studies examining the relationship between hippocampal atrophy and disruption of its efferent pathway (i.e., the fornix bundle) in both Mild Cognitive Impaired (MCI) and AD subjects have been more consistent (Pievani et al., 2010; Firbank et al., 2011; Lee et al., 2012; Mielke et al., 2012; Zhuang et al., 2012). In these studies, fornix microstructural alteration was significantly associated with reduced hippocampal volume in both MCI and AD subjects, leading the authors to hypothesize that concomitant alterations of these structures may reflect neurodegenerative processes occurring during AD as well as constituting a specific hallmark of the disease.

Morphological changes of the brain also occur during healthy aging and age-related changes of the Gray Matter (GM), including hippocampal atrophy (Scahill et al., 2003; Raz et al., 2004; Du et al., 2006) and WM changes (Salat et al., 2005a,b; Lebel et al., 2008; Pagani et al., 2008; Lee et al., 2009; Jang et al., 2011; Lebel et al., 2012; Sala et al., 2012). However, at the cellular level, hippocampal atrophy described in elderly subjects was not related to neuronal death as it was in AD, but rather with a loss of dendritic architecture (West et al., 1994; Hof and Morrison, 2004). For this reason, it is less certain that hippocampal atrophy would have an impact on its efferent and afferent fibers in the context of aging than in neurodegenerative diseases such as AD. Moreover, a major focus of neuroclinical research concerns the discrimination of healthy aging from pathological changes that occur during the early stage of the neurodegenerative disease. Alterations of the limbic system during healthy aging have been examined by few investigations to date, and their findings have been based on relatively small samples and without consideration of GM (Yassa et al., 2010; Rogalski et al., 2012).

In the present study, we hypothesize that the limbic system presents combined gray and white matter structural alterations during healthy aging. We used structural MRI covariance analysis for the description of concomitant hippocampus and WM alterations for determining limbic system damage in healthy aging. Participants were drawn from a large community-dwelling population of older individuals without dementia or MCI. To exclude MCI subjects, we used verbal episodic memory cut-offs previously established for their predictive values of dementia (Sarazin et al., 2007). Whereas volumetric GM assessment was performed on conventional T1 scans, the assessment of WM integrity was performed through exploration of FA parameter computed from DTI images. In a first analysis, age-related effects on hippocampal fractions (corresponding to the ratio between hippocampal volumes and total intracranial volumes) and on FA parameters were described for our sample of older subjects. Statistical analyses of FA parameters were performed in two ways: using a voxel-based analysis (Tract Based Spatial Statistical, TBSS) and region of interest (ROI) analyses based on cingulum and fornix bundles, major afferent and efferent pathways of the hippocampus. The TBSS analysis allowing exploration of whole WM was chosen since the limbic system is an extended network. In a second analysis, the direct relationship between hippocampal fractions and WM integrity were assessed by partialling out the effects of age (to take into account the shared variance due to age on both parameters). We hypothesize that subjects having the same age but presenting the lowest hippocampal volumes should also present the lowest FA values specifically on the connecting tracts of the hippocampus, and independently of the global effects of age on gray and white matter. Finally, to provide information on the sequential relationship between the alterations of these brain structural parameters, a recursive regression procedure was performed.

## Materials and methods

### Subjects

In this study, the participants were a subset of the AMI (Agrica-MSA-IFR de Santé Publique, Aging Multidisciplinary Investigations) cohort, an epidemiological study conducted in residents of agricultural communities (Peres et al., 2012). The AMI study started in 2007 and included 1002 older individuals who were at least 65 years old at baseline, and living in rural areas. These individuals were randomly recruited from the databases of the Farmer Health Insurance System (MSA, Mutualité Sociale Agricole). A complete battery of neuropsychological tests was administrated by a neuropsychologist at baseline and at follow ups every two years. Between March 2009 and March 2011, an MRI examination was proposed to a subsample of participants, acquiring 316 MRI exams in right-handed subjects. The study was approved by the institutional human ethics review board and all individuals in the sample provided written informed consent to participate.

Before data processing, MRI data was checked in order to discard major acquisition artifacts (*n* = 58) and major cerebral pathologies (tumor, stroke, severe WM pathologies; *n* = 55). Cerebral pathologies were detected by an experienced neuroradiologist and severity of leucoaeriosis was evaluated by two trained operators on FLAIR scans according to the Fazekas rating scale (Fazekas et al., 1987). Thirty-four subjects presenting severe leucoaeriosis (rank of 3 on the Fazekas scale) were excluded from the analyses. Since we were interested in structural changes occurring in the limbic system during healthy aging, subjects with diagnoses of dementia (according to the DSM-IV criteria; *n* = 3), global cognitive deficits measured by the Mini Mental State Examination (MMSE < 25, *n* = 17), and low performances at the Free and Cued Selective Reminding Test (FCSRT, *n* = 29) (Grober et al., 1988) were excluded. According to a previous study (Sarazin et al., 2007), verbal episodic memory is able to discriminate patients in the prodromal AD phase from those with MCI with good specificity and sensitivity. The cut-off performances applied were 17 for free recall and 40 for total recall. Subjects presenting missing MMSE or FCSRT data were also excluded from the analysis (*n* = 25). A total of 129 healthy aging subjects were included in the final sample. The second neuropsychological follow-up indicates that our sample does not include incidents cases of AD, suggesting that our MRI analysis was not performed on MCI fast converter subjects.

### MRI acquisition

MRI scans were obtained using an ACHIEVA 3T scanner (Philips Medical System, Netherlands) with a SENSE 8-channel head coil. Anatomical high resolution MRI volumes were acquired in transverse plan for each subject using a 3D MPRAGE weighted-T1 sequence with the following parameters: TR = 8.2 ms, TE = 3.5 ms, 7-degree flip angle, FOV 256 × 256 mm^2^ to cover the whole brain, yielding 180 slices, no gap, voxel size 1 × 1 × 1 mm^3^. Two diffusion-weighted images with opposite polarity, allowing elimination of diffusion imaging gradient cross-terms, were performed using a spin echo single shot EPI sequence with the following parameters: TR = 7646 ms, TE = 60 ms, 90-degree flip angle, FOV 224 × 224 mm^2^, yielding 60 slices, no gap, voxel size 2 × 2 × 2 mm^3^. One b0 image was acquired and diffusion gradients were applied in 21 non-collinear directions (*b*-value = 1000 s/mm^2^). To increase signal-to-noise ratio, the sequence was repeated in two successive runs for each polarity. All acquisitions were aligned on the anterior commissure-posterior commissure plan (AC-PC). For qualitative clinical readings, fluid-attenuated inversion recovery (FLAIR) images were also obtained with the following parameters: TR = 11000 ms, TE = 140 ms, TI = 2800 ms, FOV 230 × 172 mm^2^, yielding 24 slices, gap of 1 mm, voxel size 0.72 × 1.20 × 5 mm^3^. The total scan duration was 38 min.

### MRI analysis

#### Cerebral volumes

Cerebral volumetric assessment was performed using Voxel-Based Morphometry method (VBM) (Ashburner and Friston, 2000; Good et al., 2001). Firstly, the MRI images were spatially normalized and then segmented on their intensity distribution and spatial information derived from the ICBM prior probability maps. The unified segmentation model was extended in the VBM5 toolbox (C. Gaser; http://dbm.neuro.uni-jena.de/vbm) by applying a Hidden Markov Random Field model (Ashburner and Friston, 2005). Secondly, we applied a so-called “modulation” to each cerebral partition image. During this step, the voxel intensity of the segmented images was adjusted for the strength of deformation derived from the nonlinear spatial normalization process (Jacobian determinants). For each subject, GM, WM and CSF volumes were computed by multiplying the voxel value by the voxel size of acquisition and summing the results for all of the voxels. Total Intracranial Volume (TIV) was computed as the sum of the GM, WM, and CSF volumes.

#### Hippocampal volumes

Segmentations of bilateral hippocampi and volume estimations were performed using FIRST (Patenaude et al., 2007, 2011), part of FMRIB's Software Library (FSL) (Oxford Centre for Functional MRI of the Brain). Linear registration was performed in two stages. In the first stage, the 3D MPRAGE weighted-T1 images were registered to the non-linear Montreal Neurological Institute (MNI) 152 standard space using affine transformation with 12 degrees of freedom (DOF). Using a subcortical mask defined in MNI space, the second stage (initialized by the result of the first stage) achieves a more accurate and robust registration (12 DOF) to the MNI152 template. A segmentation based on shape models and voxel intensities was then performed using an MNI subcortical mask of hippocampus. Finally, a boundary correction method was used to determine which boundary voxels belong to the structure. Subsequently, hippocampal volumes were calculated. All images of the segmented hippocampi were visually checked for errors in registration and segmentation stages. The same procedure was applied to extract volumes of caudate nuclei, which were used as a control region.

Since preliminary analysis gave similar results considering right and left hippocampal volumes separately, right and left hippocampal volumes were averaged for statistical analyses. Hippocampal fraction (hippocampal volume/TIV) was used here as an index of hippocampal atrophy. In the same manner, volumes of control regions (global GM and caudate nuclei volumes) were normalized using the TIV.

#### FA maps

DTI images were processed with FMRIB Software Library (FSL 4.1.9, http://www.fmrib.ox.ac.uk/fsl). For each subject, diffusion-weighted images were coregistered to the reference volume b0 with an affine transformation and were corrected for motion and eddy current distortions. Brain Extraction Tool (BET) was applied to eliminate voxels not associated with the brain. DTI-data were then averaged and a FA map was computed by fitting a tensor model to the raw diffusion data.

TBSS pipeline was then used on the FA maps. Nonlinear transformations were applied to register individual FA images on the FMRIB58-FA standard space image. A mean FA was generated using all the registered individual FA maps. The resulting mean FA image was subsequently thinned to create the mean FA skeleton which corresponds to the center of all tracts common to all subjects. A threshold FA value of 0.2 was applied to limit the effects of miswarping across subjects and to reduce inclusion of voxels that are likely to be composed of multiple tissue types. Finally, each subject's FA map was warped to the skeleton by searching for maximum FA values perpendicular to the skeleton.

#### Fornix and cingulum ROIs

ROIs of fornix and cingulum were created with binary masks based on the JHU ICBM-DTI-81 WM labels atlas (Mori et al., 2008). Intersections between anatomical masks and the skeleton (previously created with TBSS) were used in order to extract mean FA values in these regions. Right and left diffusion values were averaged for statistical analyses.

### Statistical analyses

#### Age effects

The effects of age on hippocampal fraction were assessed using a linear regression model. Age was added into a model considering hippocampal fraction as the dependent variable and including gender and level of education as covariates. This analysis was performed on SPSS package (18.0.0, SPSS Inc.) and a *p*-value < 0.05 was considered statistically significant.

The effects of age on WM integrity were assessed in two different analyses. In the whole brain analysis, age was regressed onto FA maps in a model adjusted for gender and level of education. Permutation-based statistics with 5000 permutations and threshold-free cluster enhancement (TFCE) (Smith and Nichols, 2009) were used for statistical inference with a threshold of *p* < 0.05 corrected for multiple comparisons. In the ROI analysis, age was added into a model considering fornix or cingulum FA values as the dependent variable and included gender and level of education as covariates. This analysis was performed on SPSS package (18.0.0, SPSS Inc.) and a *p*-value < 0.05 was considered statistically significant.

#### Relationship between hippocampal volume and WM microstructural integrity

The relationship between hippocampal fraction and WM integrity was first assessed in a whole brain analysis. In the TBSS analysis, hippocampal fractions were regressed onto FA maps in a model adjusted for age, gender and level of education. Permutation-based statistics with 5000 permutations and TFCE with a threshold of *p* < 0.05 corrected for multiple comparisons were used for statistical inference. Subsequently, ROI analyses were performed using linear regression model on SPSS package. Hippocampal fractions were added into a model considering fornix or cingulum FA values as the dependent variable and including age, gender and level of education as covariates. In a second step, the relationship between hippocampal fraction and WM microstructural integrity was reassessed by adding WM fractions (WM volume normalized by the TIV) as covariates into the previous model including age, gender and level of education as covariates. A similar analysis was performed for caudate nuclei and global GM fractions used here as control regions and were sequentially substituted in the model to hippocampal fractions. A *p*-value < 0.05 was considered statistically significant.

Finally, to provide evidence for a temporal relationship between hippocampal damage and fornix damage, we performed recursive regression procedure with age as the dependent variable (Mormino et al., 2009). Generally speaking, a variable may be considered as a mediator if it influences the relationship between the independent variable and the dependent variable. As a result, when the mediator and the independent variable were included concomitantly in a regression analysis model, the effect of the independent variable is reduced and the effect of the mediator remains significant. A *p*-value < 0.05 was considered statistically significant.

## Results

The mean age of participants was 73.9 years old (*SD* = 4.8 years). As demonstrated by Table 1, the sample presented a mean MMSE score of 27.6 (*SD* = 1.41).

**Table 1:** **Characteristics of the sample (*n* = 129)**.

Age: mean (*SD*)	73.9 (4.8)
Female sex: No. (%)	61 (47.3)
Level of education^*^, No. (%)	
1	40 (31)
2	47 (36.4)
3	42 (32.6)
MMSE score: mean (*SD*)	27.6 (1.41)
TIV (mm^3^): mean (*SD*)	1463.10^3^ (0.16)
White matter volume (mm^3^): mean (*SD*)	423.10^3^ (0.05)
Hippocampal volume (mm^3^): mean (*SD*)	3591 (375)
Caudate nuclei volume (mm^3^): mean (*SD*)	3334 (437)
Fornix FA value: mean (*SD*)	0.43 (0.045)
Cingulum FA value: mean (*SD*)	0.47 (0.033)

* *1: primary school without diploma*,

*2: primary school validated with diploma*,

3: short secondary school, long secondary school and university level.

### Effects of age

#### Hippocampal volumes

The mean hippocampal volume was 3591 mm^3^ (*SD* = 375 mm^3^). Age was negatively correlated with hippocampal fractions (*R*^2^ = 0.320, β = −0.322, *t* = −4.102, *p* < 0.001, adjusted for gender and level of education).

#### WM integrity

**Figure 1:**
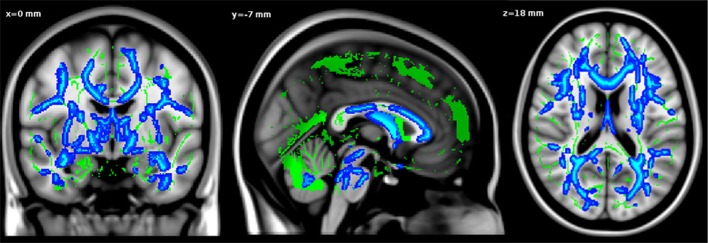
**Negative relationship between age and FA values.** Significant voxels are shown in blue. The results are displayed at *p* < 0.05, TFCE corrected and overlaid simultaneously on the mean FA skeleton (green) and the MNI template.

The whole brain TBSS analysis revealed that age was negatively associated to FA values in almost the whole WM skeleton, including fornix and cingulum bundles (*p* < 0.05, TFCE corrected, adjusted for gender and level of education) (Figure 1).

As demonstrated by Table 1, the mean fornix FA was 0.43 (*SD* = 0.045) and the mean cingulum FA was 0.47 (*SD* = 0.033). In accordance with whole brain analysis, ROI analysis revealed a negative correlation between age and fornix FA values (*R*^2^ = 0.165, β = −0.341, *t* = −3.914, *p* < 0.001, controlled for gender and level of education). A negative correlation was also observed between age and cingulum FA values (*R*^2^ = 0.156, β = −0.308, *t* = −3.520, *p* = 0.001, adjusted for gender and level of education).

### Relationship between hippocampal volume and WM integrity

#### TBSS analysis

**Figure 2:**
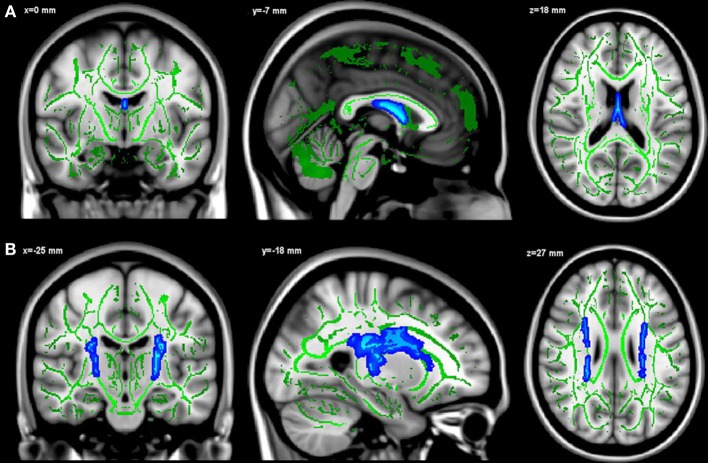
**Positive (A) and negative (B) relationships between hippocampal fractions and FA values.** Significant voxels are shown in blue. The results are displayed at *p* < 0.05, TFCE corrected and overlaid simultaneously on the mean FA skeleton (green) and the MNI template.

Only FA values in a cluster restricted to the fornix were positively associated to hippocampal fractions (*p* < 0.05, TFCE corrected, adjusted for age, gender, level of education) (Figure 2A). Surprisingly, the whole-brain analysis revealed that hippocampal fractions were also negatively associated to FA values at the level of cortico-spinal and arcuate fasciculi (*p* < 0.05, TFCE corrected) (Figure 2B).

#### ROI analyses

**Figure 3:**
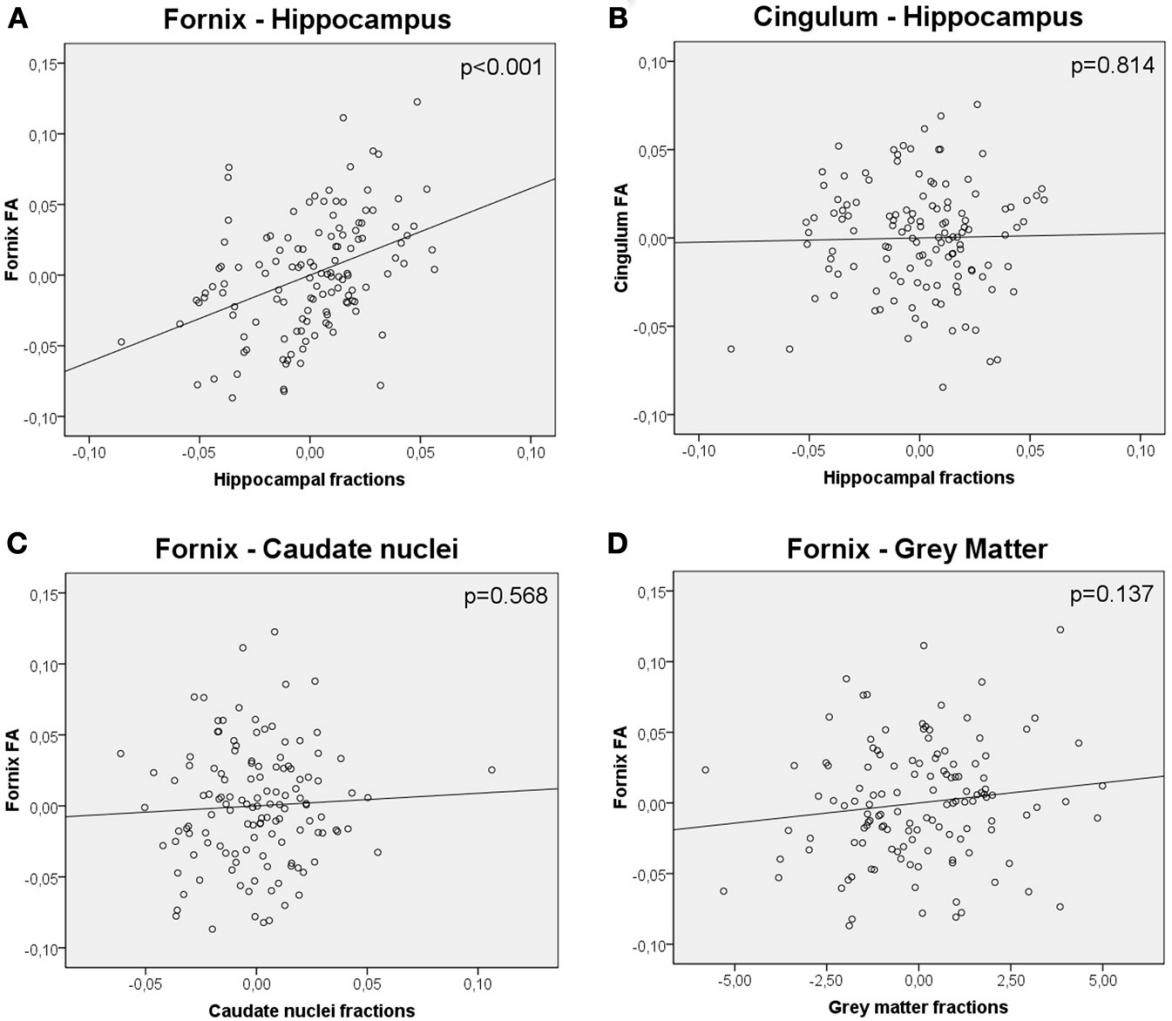
**Partial regression plots showing the relationship between (A) FA values of the fornix and hippocampal fractions (B) FA values of the cingulum and hippocampal fractions (C) FA values of the fornix and caudate nuclei fractions (D) FA values of the fornix and gray matter fractions.** Variables in each plot were adjusted for age, gender and level of education. The relationship between FA values of the fornix and hippocampal fractions was significant (*p* < 0.001), whereas the others were not.

As with TBSS analysis, a significant positive association was observed between hippocampal fractions and fornix FA values (*R*^2^ = 0.299, β = 0.444, *t* = 4.876, *p* < 0.001, adjusted for age, gender and level of education) (Figure 3A). When global WM fractions were added to the model, the association between hippocampal fractions and fornix FA values remained significant (*R*^2^ = 0.428, β = 0.233, *t* = 2.536, *p* = 0.012). No significant relationship between hippocampal fractions and cingulum FA values was observed (*R*^2^ = 0.157, β = 0.024, *t* = 0.235, *p* = 0.814) (Figure 3B). When global GM and caudate fractions were sequentially substituted in the model to hippocampal fractions, no significant association was observed (Figures 3C,D).

In a model including hippocampal fractions as a predictor of age, hippocampal fractions presented a significant effect (*R*^2^ = 0.079, β = −0.281, *t* = −3.300, *p* = 0.001). In a model including fornix FA values as a predictor of age, fornix FA values also presented a significant effect (*R*^2^ = 0.116, β = −0.340, *t* = −4.074, *p* < 0.001). Finally in a model including hippocampal fractions and fornix FA values as simultaneous predictors of age, hippocampal fractions lost significance (*R*^2^ = 0.132, β = −0.147, *t* = −1.529, *p* = 0.129) whereas FA fornix values remained significant (*R*^2^ = 0.132, β = −0.266, *t* = −2.766, *p* = 0.007) suggesting a mediation effect of the FA fornix values in the relationship between hippocampal fractions and age.

## Discussion

In this study of a large sample of healthy aging subjects, we observed age-related changes of hippocampal volumes as well as white matter FA values. Secondly, we observed that hippocampal fraction was associated with reduced FA value at the level of the fornix bundle, in a regression analysis partialling out for the effects of age. This analysis indicates that the relationship observed here between hippocampal volumes and FA fornix values is not only shared variance due to indirect concomitant effect of age on both type of tissue. Reinforcing this assertion, hippocampal volume was not associated with changes at the level of another main connecting tract, the cingulum bundle, which nevertheless presents age-related modifications. The ROI analysis confirmed the specificity of this association between hippocampal volume and fornix microstructure integrity in healthy aging. Moreover, the findings suggest that a part of the effects of age on hippocampal volumes is in fact mediated by fornix integrity, supporting the hypothesis that healthy aging is associated with the structural disorganization in the limbic system spreading from connecting fibers to the hippocampus.

### Age-related damage

Our sample of older adults presents age-related changes at different levels of the brain. Older subjects present a lower hippocampal fraction, which could reflect an atrophy of this structure, and present a decrease of FA values across the whole WM tracts. These results are in accordance with previous studies in late adulthood. For the hippocampus, the effects related to age observed here could be related to later acceleration of atrophy as described in lifespan studies (Raz et al., 2004; Allen et al., 2005; Jernigan and Gamst, 2005; Fjell et al., 2009). Studies based on DTI clearly demonstrate that brain still matures on its WM part until the age of 40, which clearly enters the range of adulthood. Nonlinear associations between age and DTI parameters across the adult lifespan have been demonstrated depending on the tract considered. However, when healthy older subjects (over 65) are considered, FA decreased consistently with age in all tracts (Head et al., 2004; Pfefferbaum et al., 2005; Salat et al., 2005a,b; Lee et al., 2009; Yassa et al., 2010; Jang et al., 2011; Kantarci et al., 2011; Metzler-Baddeley et al., 2011, 2012; Rogalski et al., 2012; Sasson et al., 2013) as observed here.

### Relationship between hippocampal atrophy and fornix damage

Aging is associated with brain structural alterations occurring together in both gray and white matter, but their relationship within the neuronal network has not been extensively studied. We observed that hippocampal atrophy is associated with alterations of fornical efferent fibers controlling for the effects of age on both types of tissue. Through a structural covariance study, we demonstrated a direct relationship between two structural alterations occurring during healthy aging at the level of the limbic system. In other words, in subjects of the same age, the one who has the lowest hippocampal fraction will also present lowest FA values at the level of the fornix bundle. The findings suggest that a part of the effects of age on the fornix, previously described in lifespan studies (Salat et al., 2005a,b; Lebel et al., 2008; Pagani et al., 2008; Lee et al., 2009; Jang et al., 2011; Lebel et al., 2012; Sala et al., 2012), is related specifically to hippocampal atrophy and not only to age-related global WM atrophy. Two sources of evidence support the specificity of this observed relationship. First, hippocampal atrophy was related to fornix FA values but not to cingulum FA values, thereby counteracting a global nonspecific effect. Second, when WM atrophy was added as a covariate, the relationship between hippocampal atrophy and fornix FA value remained significant. To date, very few studies have examined limbic network integrity during aging (Lee et al., 2012; Zhuang et al., 2012). Among them, Zhuang et al. (2012) demonstrated a relationship between normalized hippocampal volumes and FA values of the fornix. On the contrary, Lee et al. (2012) failed to demonstrate a correlation between volumes of the hippocampal CA1 and subiculum subfields with fornix FA values in a relatively large sample of older adults (*n* = 96). This apparent discrepancy could be explained by technical issues in that Lee et al. (2012) used a 6 directional DTI performed on a 1.5T scanner which may have contributed to poor sensitivity, compared to more than 21 directional DTI performed on 3T scanner for the investigation by Zhuang et al. (2012) as well as the current study. The question also persists concerning the cellular mechanism involved in age-related fornix bundle deterioration. The decrease of FA observed could be due simply to a demyelinization process, or to the degeneration of nerve fibers as described recently at the level of the fornix bundle in aging monkeys (Peters et al., 2010). Whereas causality between the alterations of these two parts of the network is demonstrated here, the direction (i.e., fornix fibers degeneration brought about hippocampal atrophy or fornix fibers degeneration induced hippocampal atrophy) could not be assessed robustly in this transversal analysis. However, the recursive regression procedure performed here strongly suggests that hippocampal atrophy in healthy aging could result from a disconnection process.

From a functional point of view, several previous studies on humans and monkeys have highlighted the importance of structural preservation of the fornix in episodic memory performances (Gaffan et al., 1991; Gaffan, 1992; Parker and Gaffan, 1997; Aggleton et al., 2000; Tate and Bigler, 2000; Gaffan, 2001; Zahajszky et al., 2001). More recently, researchers have found that greater microstructural integrity of the fornix is related to better recollection performance (Tsivilis et al., 2008; Rudebeck et al., 2009; Metzler-Baddeley et al., 2011; Zhuang et al., 2012), and Metzler-Baddeley et al. (2012) proposed that fornix alteration could underlie, at least in part, the age-related memory decline. In the present study, we did not observe a significant correlation between fornix FA values and verbal episodic memory performance (data not shown). The absence of functional outcomes of axonal pathway damage in the limbic network may be explained by the fact that subjects were selected on the basis of high cognitive performances (particularly for episodic memory) in order to exclude subjects with early pre-symptomatic dementia. In addition, the sample may have been more likely to use compensation strategies leading to the preservation of normal memory performance despite axonal damage. It is possible that this compensatory phenomenon remained efficient when limbic network changes are minor as in our sample of healthy aging subjects, but that saturation of this compensatory process may occur with the progression of age-related network damage or ongoing neurodegeneration in the pre-dementia phase. Another explanation could be that impaired memory performance classically observed in healthy older subjects relies on executive function impairment and consequently on dysfunction of frontal networks rather than on dysfunction of the limbic system (Bernard et al., 2007; McDonough et al., 2013).

No relationship has been shown between hippocampal atrophy and DTI metrics of the cingulum bundle in healthy aging subjects. This lack of association could be explained by the topographical organization of the cingulum bundle which is complex and heterogeneous, rendering measurement difficult. When we considered its two major components separately in a supplementary analysis, we did not observe a relationship between hippocampal atrophy with either the parahippocampal cingulum or medial cingulum (data not shown). Whereas some studies demonstrated a relationship between hippocampal atrophy and the cingulum bundle in neurodegenerative diseases such as AD (Xie et al., 2005; Villain et al., 2008; Choo et al., 2010; Sexton et al., 2010; Villain et al., 2010; Agosta et al., 2011), we did not observed such a relationship in healthy aging subjects suggesting that it was selectively associated with neurodegenerative process.

### Methodological considerations

The cross-sectional design of our study did not allow us to dissociate the underlying mechanisms of weak hippocampal fraction, which may therefore reflect either age-related hippocampal atrophy or pre-existing low hippocampal volume determined early in life. To deal with this problem, 31 scans of subjects in the lowest quartile for hippocampal fraction were visually inspected by 2 raters (AP and GC) to scale hippocampal atrophy while blinded to volumetric assessment. Subjects presenting a low hippocampal fraction were described as presenting hippocampal atrophy by blinded observers (Supplementary Data 1 presents two scans to illustrate low and high hippocampal fractions). Moreover, the significant association between age and hippocampal fractions observed in our analysis supports the atrophy hypothesis rather than an interindividual variability in volume that is determined early in life. Thus, according to these observations, hippocampal fraction is taken as an index of hippocampal neural alteration whether it represents neural loss or neural shrinkage.

It has been shown that age-related GM and WM atrophy may cause partial volume effects from the CSF, particularly in fornix bundle which lies along CSF spaces (Concha et al., 2005; Jones and Cercignani, 2010). Indeed, age-related brain tissue loss can lead to CSF partial volume effects, which may lead in turn to an underestimation of diffusion MRI parameters (Ge et al., 2002). In our sample, the mean FA value of the fornix was 0.43, which is in the range of previously published study (Concha et al., 2005). Moreover, two methodological considerations argue against this potential source of error. First, when WM atrophy was added as a covariate, the relationship between hippocampal atrophy and fornix FA value remained significant. Second, we performed the same analysis using a group specific template (Keihaninejad et al., 2012), and the result of this whole brain analysis is very similar indicating a significant relationship between hippocampal fractions and fornix FA values. More importantly, to evaluate the reliability of our assessment, extracted FA values of the fornix were regressed onto GM volumetric maps. This analysis revealed an association with both the source of the fornix, i.e., the hippocampus and its main projection site, i.e., mammillary bodies (Loftus et al., 2000; Tsivilis et al., 2008), demonstrating reliable assessment of FA (Supplementary Data 2). Finally, recent studies have demonstrated that FA values, in particular the index used in this present study, were less prone to CSF contamination than diffusivity values (axial, radial and mean diffusivities) (Pasternak et al., 2009; Vos et al., 2011). Taken together, these elements argue against the interpretation of our findings are being due to a CSF partial volume effect.

Surprisingly, our voxel-based analysis indicates that hippocampal fraction is related to an increase of FA in cortico-spinal and arcuate fasciculi. Increase of FA in regions including multi fiber population (as it is the case for the above regions) could be due either to an increase of coherence of the main tract or to a decrease of coherence of the minor fibers crossing the main tract. To interpret this increase of FA, we also performed an analysis using the mode of anisotropy (MO) (Supplementary Data 3), an additional diffusion index which partially resolves the problem of crossing fiber regions (Ennis and Kindlmann, 2006). MO specifies the shape of the diffusion tensor from planar to linear. Planar shape reflects diffusion in a region holding two roughly equal fiber populations whereas linear shape reflects diffusion in a region where one fiber population orientation predominates. Regression analysis performed on MO maps indicates that lowest hippocampal fractions were significantly associated with a more linear shape of the diffusion tensor at the level of the cortico-spinal and of the arcuate fasciculi. The co-localized increase of FA and MO values suggests a global decrease of coherence of fibers crossing the cortico-spinal and the arcuate fasciculi with a preservation of these two main tracts. In the same way, a recent study found that the co-localized increase of FA and MO was explained by a relative preservation of one population compared with others in a region of crossing fibers (Douaud et al., 2011). In addition, ROI analysis indicated that when global WM fractions were added as a covariate, the relationship between hippocampal fractions and FA values in the posterior limb of the internal capsule (a part of the corticospinal tract) lost significance. This latter result suggests that the global degeneration of fibers crossing the cortico-spinal tract and the arcuate fasciculi is related to global WM atrophy rather than to hippocampal atrophy. Hippocampal fractions and FA values of the cortico-spinal and the arcuate fasciculi are together linked to WM fractions; they share variance related to age that drives the observed relationship.

As AD has been shown to involve insidious neurodegenerative processes over many years, even the use of a FCSRT cutoff score to identify at-risk AD subjects would not be sufficient to exclude the possibility that subclinical AD pathology might underlie the relationship between hippocampal atrophy and fornix integrity disruption The second neuropsychological follow-up of the study indicates that the sample used in our analysis did not include any incident AD cases. However, consideration of this important issue requires a substantial follow-up period of up to decade that is not yet available on this cohort.

## Conclusion

The present findings demonstrate that limbic system alterations during healthy aging involve the hippocampus, the fornix bundle, and the cingulum bundle. Moreover, age-related hippocampal atrophy may be directly associated to the loss of microstructural integrity of the fornix bundle but not of the cingulum bundle. Since the fornix is mainly constituted of efferent fibers from the hippocampus, age-related damage of the fornix could impact hippocampal structure through retrograde processes. The temporal relationship between these structural alterations could be firmly assessed on longitudinal data. Whatever, limbic system alterations at its fornix part appeared during healthy aging and should not be considered as a specific hallmark of the neurodegenerative process occurring during AD.

### Conflict of interest statement

The authors declare that the research was conducted in the absence of any commercial or financial relationships that could be construed as a potential conflict of interest.
