# Supplementary material for: Structural hippocampal network alterations during healthy aging: a multi-modal MRI study
Source: Front Aging Neurosci. 2013 Dec 5;5:84. doi: 10.3389/fnagi.2013.00084 (PMC3852215; doi:10.3389/fnagi.2013.00084)
Supplement: Supplementary file 1 [file DataSheet1.ZIP › 66547_Pelletier_Data_Sheet_3.pdf]

Supplementary Data 3: Focusing on the crossing fiber regions: understanding increased diffusion anisotropy in cortico-spinal and arcuate fasciculi.

Surprisingly, TBSS analysis revealed that hippocampal fractions were also significantly associated to high FA values at the level of cortico-spinal and arcuate fasciculi ($p<0.05$, TFCE corrected). As interpretation of FA changes is difficult in regions containing multi-fiber populations, we used the mode of anisotropy (MO), an additional diffusion index sensitive to diffusion changes in fiber populations (Ennis and Kindlmann, 2006). MO is a diffusion index, orthogonal to FA, which specifies the shape of the diffusion tensor and thereby provides complementary information to FA. It varies from -1 to +1 as the shape of the diffusion tensor ranges from planar (e.g. in regions of crossing fibers with two roughly equal fiber populations) to linear (e.g. in regions where one fiber population orientation predominates).

Regression analysis performed on MO maps indicates that hippocampal fraction was significantly associated with a more linear shape of the diffusion tensor at the level of the cortico-spinal and arcuate fasciculi ($p<0.05$, TFCE corrected) (Supplementary Figure 4). Accordingly, ROI analysis indicates a significant negative relationship between hippocampal fractions and FA values in the posterior limb of the internal capsule, a part of the corticospinal tract ($R^2=0.116$, $\beta=-0.318$, $t=-3.108$ and $p=0.002$). However, when WM fractions were added to the model, the relationship lost significance ($R^2=0.147$, $\beta=-0.214$, $t=-1.909$ and $p=0.059$).

Our voxel-based analysis indicates that hippocampal fraction is related to an increase of FA values in cortico-spinal and arcuate fasciculi, concomitantly to an increase of MO values. The latter indicates that one fiber population orientation predominates in these regions when hippocampal fraction decreases. The co-localized increase of MO and FA values suggests that hippocampal atrophy is associated to a global degeneration of the fibers crossing the cortico-spinal and arcuate fasciculi and a relative preservation of the fibers constituent these two major fasciculi. In addition, ROI analysis indicated that when WM fractions were added as a covariate, the relationship between hippocampal fractions and FA values in the cortico-spinal tract losts significance, suggesting that the relationship observed in this WM region is due to residual shared variance related to age.

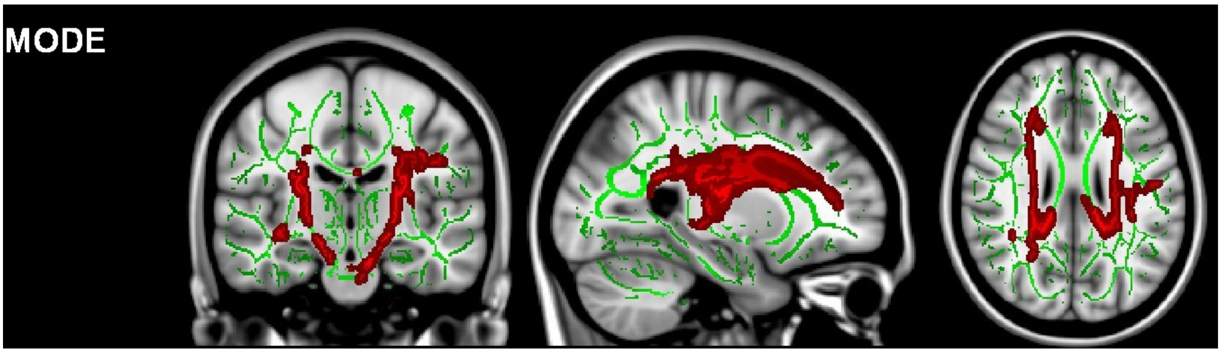

Supplementary Fig.3. Negative relationship between hippocampal fractions and MO values (red). Regions holding multi-fiber populations were highlighted. The increase of MO values indicates that in these regions of crossing fibers, a single fiber population predominates when the hippocampal fraction decreases. The results are displayed at $p<0.05$, TFCE corrected and overlaid simultaneously on the mean FA skeleton (green) and on the MNI template.
