# Supplementary material for: Structural hippocampal network alterations during healthy aging: a multi-modal MRI study
Source: Front Aging Neurosci. 2013 Dec 5;5:84. doi: 10.3389/fnagi.2013.00084 (PMC3852215; doi:10.3389/fnagi.2013.00084)
Supplement: Supplementary file 1 [file DataSheet1.ZIP › 66547_Pelletier_Data_Sheet_1.pdf]

**Supplementary Data 1**

In order to verify the presence of atrophy in subjects presenting low hippocampal fractions, the level of hippocampal atrophy was visually inspected by two experienced raters blinded as to volumetric assessment. The Supplementary Figure 1 illustrates the scan of subject presenting a high and a low hippocampal fraction. As demonstrated by the scans, subjects with low hippocampal fractions present visible atrophy.

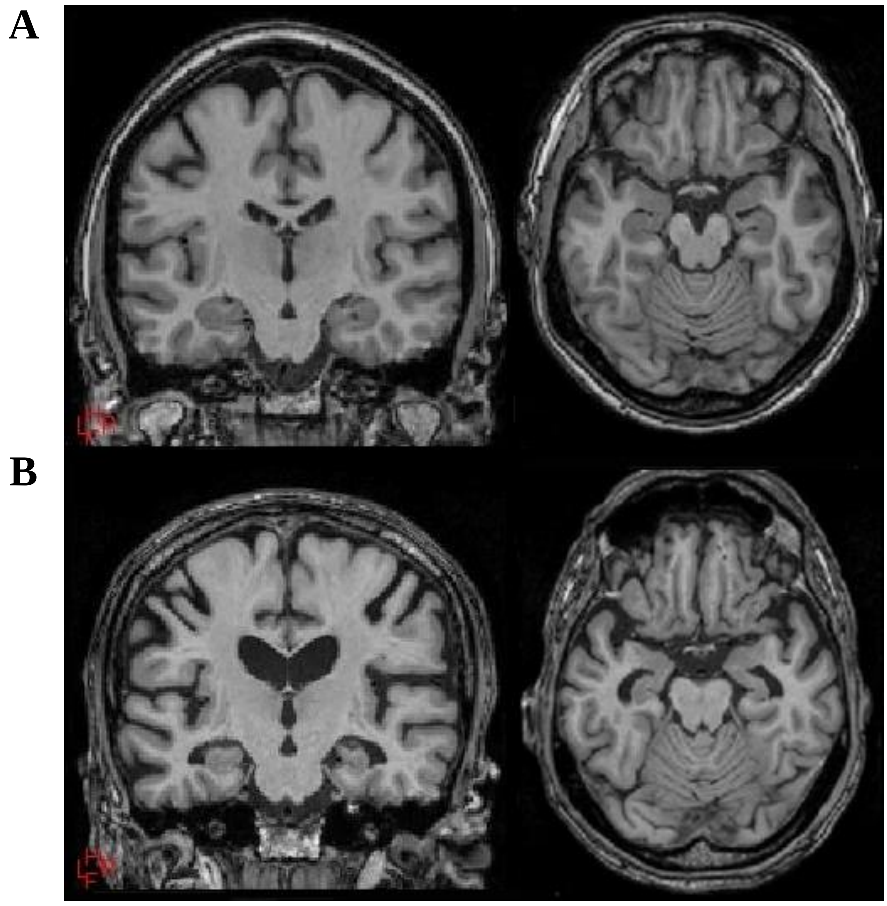


**Supplementary Fig.1.** T1 scans of individuals presenting a high (A) and a low (B) fraction of hippocampus.
