# Supplementary material for: Structural hippocampal network alterations during healthy aging: a multi-modal MRI study
Source: Front Aging Neurosci. 2013 Dec 5;5:84. doi: 10.3389/fnagi.2013.00084 (PMC3852215; doi:10.3389/fnagi.2013.00084)
Supplement: Supplementary file 1 [file DataSheet1.ZIP › 66547_Pelletier_Data_Sheet_2.pdf]

Supplementary Data 2

Due to its location and narrow dimensions, the fornix is prone to partial volume errors which can bias the estimation of FA values. In order to test the specificity of the extracted FA values, they were regressed onto GM volumetric maps. Before statistical analysis, the GM volumetric maps were smoothed using an 8-mm Full Width at Half Maximum Gaussian Kernel. The smoothed partitions were then masked with an exclusive mask of GM. This mask was built using the SPM5 Masking toolbox in order to exclude any voxel that was unlikely to belong to GM. For statistical analysis, we applied a statistical threshold of $p<0.05$ after corrections for multiple comparisons using topological False Discovery Rate (FDRp) and with a significant threshold cluster of 100 voxels.

Regression analysis performed on GM volumetric maps indicated that weak FA values were associated with weak GM volumes of the hippocampi and the mammillary bodies, the two GM regions connected by the fornix bundle (topological FDRp, $T>4.578$) (Supplementary Figure 3).This result underlies the specificity of the measurement performed here on the fornix and argue against mis-measurement due to partial volume effects.

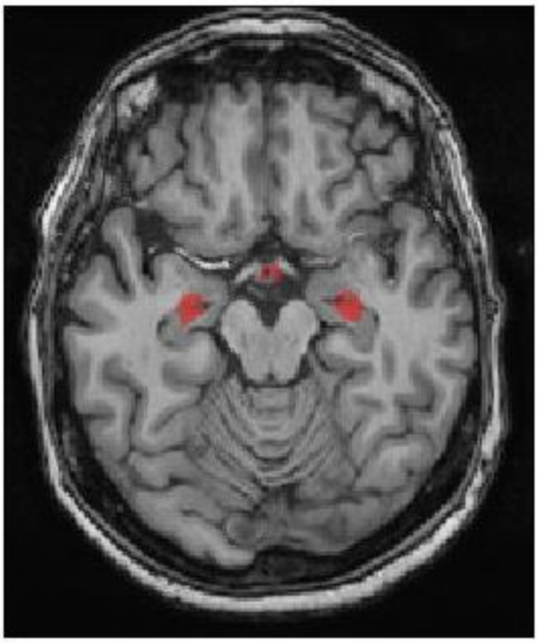

Supplementary Fig.2. Fornix FA values were regressed onto GM maps in the whole group. Voxels highlighted in red were significantly correlated to fornix FA values (Topological FDRp, $T>4.578$). Fornix FA values extracted here were correlated with volumes of two GM regions, the hippocampi and the mammillary bodies, underlying the specificity of the FA assessment.
